# Scalable fabrication of precise flexible strain sensors using organic semiconductor single crystals

**DOI:** 10.1080/14686996.2025.2451020

**Published:** 2025-01-31

**Authors:** Yoshihisa Usami, Yu Yamashita, Tomohiro Murata, Takafumi Matsumoto, Masataka Ito, Shun Watanabe, Jun Takeya

**Affiliations:** a Department of Advanced Materials Science, Graduate School of Frontier Sciences, The University of Tokyo, Kashiwa, Chiba, Japan; b Research Center for Materials Nanoarchitectonics, National Institute for Materials Science (NIMS), Tsukuba, Ibaraki, Japan; c Pi-Crystal Inc, Kashiwa, Chiba, Japan

**Keywords:** Organic semiconductor, strain sensor, chemical doping

## Abstract

Organic semiconductor (OSC) single crystals feature flexibility, solution processability, and high-mobility coherent carrier transport, which are advantageous for printed flexible electronic applications. A mechanical strain sensor is a target device whose high sensitivity and wide measurement range have been demonstrated when OSC single crystals were employed as the active channel. However, there have been limited reports on scalable fabrication of devices and reliable measurements, which limits the use of strain sensors in a wide range of applications. In this study, we present a comprehensive approach to address these issues through advanced device processing, design, and measurements. Our resistive strain sensors showed a small drift owing to the stable and effective p-type chemical doping of the OSC single crystals. A Wheatstone bridge circuit and compact lock-in amplifier were designed to accurately measure resistance changes at low noise levels. The experimental results demonstrated a substantial reduction in noise and achieved high-precision measurements with precision of ± 1.8 ppm. These results demonstrate the scalable fabrication of organic semiconductor strain sensors with high precision and reliability, which opens up the possibility of employing them in various industrial sectors.

## IMPACT STATEMENT

ppm-scale strain sensing was demonstrated using flexible sensors based on p-type doped organic semiconductor single crystals fabricated on a 10 cm square substrate.

## GRAPHICAL ABSTRACT

**Figure:**
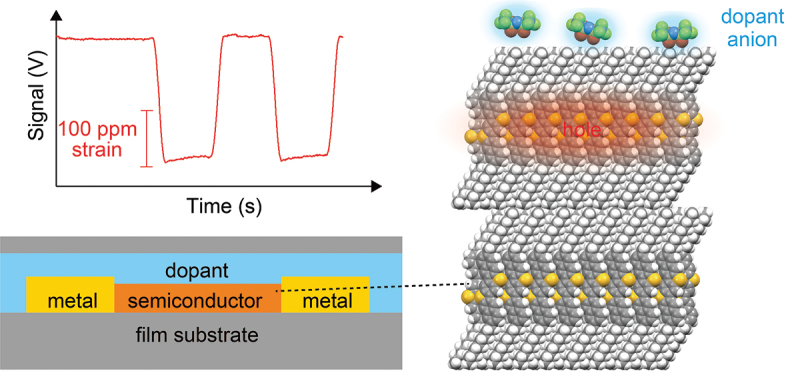

## 1. Introduction

Organic semiconductors (OSCs) are candidate materials for flexible printed electronics owing to their flexibility, solution processability, and electronic properties, which can be tuned by molecular design [1]. Single crystals of OSC [2–5] show high mobility coherent carrier transport, which is important in fabrication of reliable electronic devices. Devices to develop sensor networks, including transistors [6,7], sensors [8], and wireless communication devices such as radio-frequency identification tags [9], have been studied using OSC thin-film single crystals fabricated through solution processes [10–14]. One target sensor using intrinsically soft OSC single crystals is a strain sensor. Carrier transport in OSC single crystals is strongly influenced by molecular vibrations in the soft lattices. The lattice constant and molecular vibrations of OSC single crystals respond to applied mechanical strains, which results in reproducible responses, high sensitivity, and a wide measurement range for strain sensors based on them [8,15].

Strain sensors showing flexibility, a wide measurement range, and high sensitivity will contribute to various sectors, including robotics, infrastructure monitoring, and healthcare. Strain sensors are basic components used to measure the loads and displacements of target materials employed in broad fields, from robotics and heavy industry to microchip devices [16–19]. Signals such as vibrations are also measured using strain sensors with an appropriate device design to operate at high frequencies [20]. Flexible sensors with a wide measurement range and high sensitivity can be used in healthcare to monitor the motion of the human body. Chemomechanical sensors, which are suitable for biochemistry and healthcare, have been developed by combining strain sensors with membranes that capture chemicals [21,22]. The sensitivity of a strain sensor is expressed by the gauge factor, which is the ratio of the changes in the electronic resistance and mechanical displacement. Conventional strain sensors employing metal foils exhibit flexibility, however, their gauge factors are rather small. Inorganic semiconductors, such as silicon, exhibit high sensitivity. Although the use of thin silicon membranes has been demonstrated in mechanochemical sensors [23], they may not be suitable for flexible strain sensors that can be attached to various sensing targets. OSC single crystals show a gauge factor of approximately 20 (Table 1) and flexibility owing to their intrinsic softness and thinness, which is advantageous for achieving highly sensitive flexible strain sensors for various sensing targets.

**Table 1.** Comparison of materials for strain sensors. A: an expected value based on single crystals of another organic semiconductor, rubrene [24].

	nichrome thin film [25,26]	Si single crystal [27,28]	C9-DNBDT-NW single crystal [8]
gauge factor	2	~200	23
maximum strain	5%	0.5%	~1.5%
Young’s modulus	150 GPa	130 GPa	~10 GPa^A^
thickness	<100 nm	>1 μm	10 nm

To advance the implementation of strain sensors with OSC single crystals, the reliability of the sensors must be demonstrated in terms of drifts, noises, and precision, which has not been fully addressed to our knowledge. Organic semiconductors can be utilized as easy-to-measure resistive sensors by increasing their carrier concentrations via chemical doping [15]. Considering the trap density of states in OSCs [29], effective chemical doping also plays a key role in trap filling and decrease in noise levels. Although stable and effective doping is necessary to achieve a small drift and noise, the stability of chemical doping of OSC under ambient conditions has been rather challenging [30].

This study reports the sensor structure and measurement circuit for the practical implementation of highly sensitive and reliable strain sensors utilizing OSC single crystals. Scalable, reproducible, and stable chemical doping has been employed in OSC single crystals, where the chemical doping process is modified ion-exchange doping [31]. Strain sensing was conducted using a Wheatstone bridge, where the ppm-scale strains were successfully measured using our sensors. A compact lock-in amplifier circuit dramatically improves the signal-to-noise ratio. These findings pave the way for industrialization and the development of new applications for strain sensors using the OSC single crystals.

## 2. Experimental

**Figure 1.**
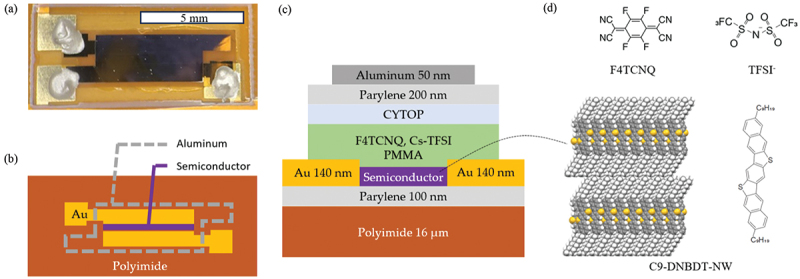
The structure of the strain sensor. (a) A photo, (b) and an illustration of the fabricated strain sensor from the top view. (c) An illustration of the fabricated strain sensor from the side view. (d) Chemical structure of the dopants and semiconductor together with its crystal structure. The channel direction, c-axis, is the horizontal direction of the panel.

The structure of fabricated strain sensors is shown in Figure 1(a-c). The employed materials are shown in Figure 1(d). For the substrate, a polyimide film from Toray-DuPont, with a thickness of 16 μm was employed. This thin thickness was chosen to accurately transmit mechanical signals of a target object to the OSC single crystal. On this substrate, 100 nm parylene and OSC single-crystal layers were fabricated. 3,11-dinonyldinaphtho[2,3-*d*:2’,3’-*d*’]benzo[1,2-*b*:4,5-*b*’]dithiophene (C9-DNBDT-NW) was employed as OSC in this study, whose molecular structure is shown in Figure 1d. The C9-DNBDT-NW single-crystal film was formed using the continuous edge casting method according to the literature, which typically forms thin films consisting of a few molecular layers [14]. After this solution process, the device was heated at 80°C in vacuum overnight. Gold electrodes were thermally deposited on the single crystals through a shadow mask. Then, chemical doping was conducted by spin coating a dopant layer, which will be explained in detail later. The OSC and dopant layers were patterned using a dry etching process. As encapsulation layers, CYTOP, parylene, and Al layers were fabricated. Three-times diluted Cytop809M was spin-coated at 2,000 rpm. The Parylene layer was 200 nm. The Al layer, which also serves as shielding by connecting to the ground line, was thermally deposited with a 100 nm thickness through a shadow mask.

Chemical doping in this study was conducted based on the ion-exchange doping [31]. In this method, electron transfer reactions between 2,3,5,6-tetrafluoro-7,7,8,8-tetracyano-quinodimethane (F4TCNQ) and OSC introduce holes into the OSC. Following this, the ion pair [OSC^•+^ F4TCNQ^•-^] and bis(trifluoromethanesulfonyl)imide (TFSI^−^) in the system undergo ion exchange reactions, where the doped OSC [OSC^•+^TFSI^−^] is the final product. Importantly, this doping method has been reported to dope the surface of C9-DNBDT-NW single crystal without changing the crystal structure [15]. In previous reports, ion-exchange doping has been conducted by immersing an OSC layer in dopant solutions or spin coating dopant solutions on an OSC layer. For the surface doping of single crystals, such a process may leave crystallites of dopants on the surface of OSC, limiting the reliability in device fabrication. In this study, PMMA was added as a binder to the dopant solution. In this case, the dopant layer of F4TCNQ, CsTFSI, and PMMA were fabricated on the OSC single crystal uniformly. The employed solution contained 0.175 wt% F4TCNQ, 0.3 wt% CsTFSI, and 2 wt% PMMA in 2,2,2-trifluoroethanol. This solution was spin-coated at 2,000 rpm for 60 s.

## 3. Results and discussion

**Figure 2.**
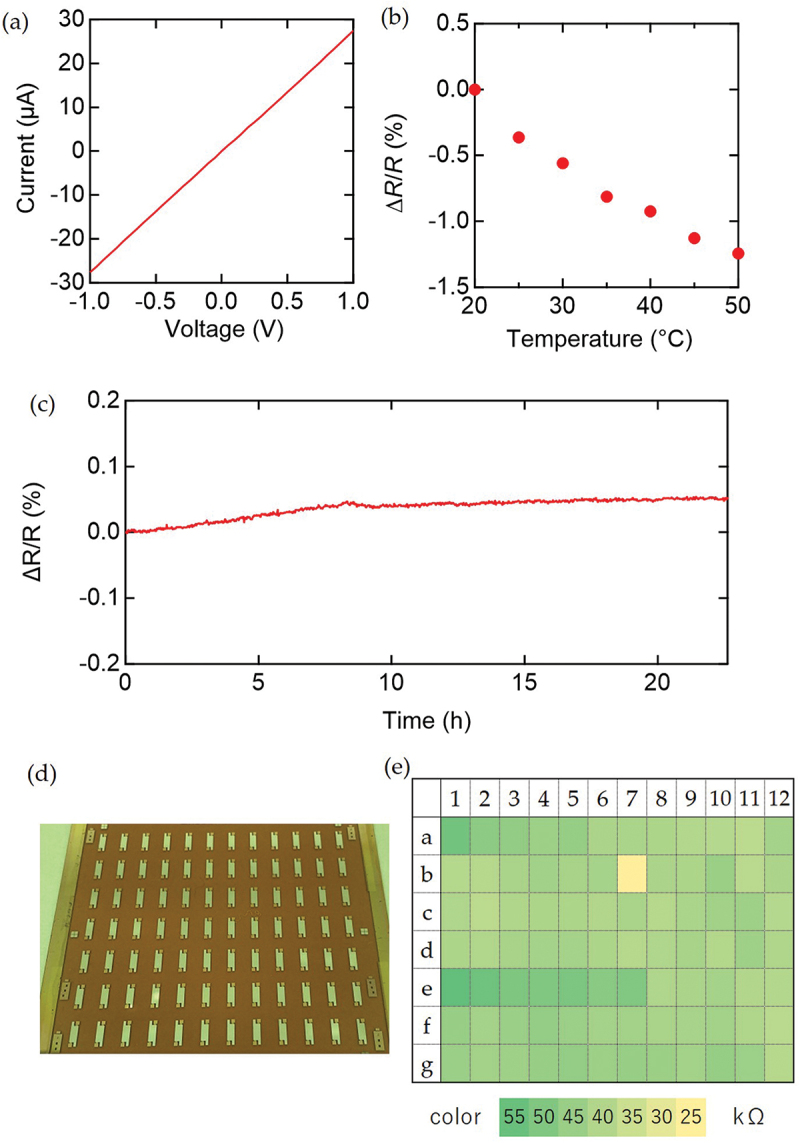
The electrical characteristics of the fabricated sensors. (a) A representative current-voltage characteristics of the fabricated sensor. (b) Temperature dependence of the resistance of the fabricated sensor. (c) The drift in resistance of fabricated sensor at 20°C in air. (d) A photo of the fabricated sensors on a 10 cm square substrate. (e) The resistance distribution of the sensors fabricated on the 10 cm square substrate.

The current-voltage characteristics of the fabricated strain sensors are shown in Figure 2(a). The resistance of the sensor was *ca*. 40 kΩ, which is reasonably low to be employed as a resistive sensor. The sheet conductivity of *ca*. 3 μS suggests that the Fermi energy is close to the valence band edge, where effective trap filling is expected [29]. This is important for achieving band transport in OSC single crystals, which leads to a reliable resistance response against mechanical strains [7,15]. Band transport in our system was also supported from the temperature dependence of the resistance measured in a temperature-controlled chamber (ESPEC SH-222). Based on the observed weak temperature dependence of the resistance (Figure 2(b)), the activation energy of the carrier transport was estimated to be 3.3 meV, which is much lower compared to the typical value for hopping carriers in organic semiconductors (~100 meV [32]). While the observed temperature dependence suggests that the sensor resistance would drift by the effect of temperature, this effect can be reduced by employing the active dummy method, for instance, which will be studied in detail in future works. The drift of the fabricated sensor was evaluated by continuously monitoring the electrical resistance at 20°C in air (Figure 2(c)). The drift in the resistance (*ΔR/R*) was a small value of 0.005% per hour for the initial 8 h, which further decreased during the 22 h of monitoring. To achieve stable resistance and doping levels in chemically doped organic semiconductors, it is advantageous to employ inert closed-shell dopant ions [31]. In this study, we employed the modified ion-exchange doping, where the dopants are mixed with PMMA serving as a medium to achieve uniform dopant film formation. Such a high stability of chemically doped OSC single crystal has not been demonstrated to our knowledge.

84 sensors with the above structure were fabricated on a 10 cm square substrate as shown in Figure 2(d). The distribution of resistance values is shown in Figure 2e. Most of the devices exhibited resistances in a reasonable range. The average resistance of this prototype batch was 42.8 kΩ, with a standard deviation σ of 4.1 kΩ, corresponding to 9.4% of the average value. Excluding the four data that are more than three standard deviations away from the mean, a standard deviation was evaluated to be 6.7%. This successful fabrication of a large number of devices owed to our scalable fabrication process of OSC single-crystal sensors using the continuous edge casting and ion-exchange doping with a PMMA binder.

To achieve precise detection of the strain signal using our sensor, a compact measurement circuit was built in this study. In strain measurements, the Wheatstone bridge circuit is commonly utilized to accurately measure small changes in resistance. This circuit enables the detection of voltage fluctuations caused by small resistance changes in the sensor. In this circuit, noise from the sensor is also amplified, which may limit the attainable reliability and detection of small signals. To decrease the noise level, a compact lock-in amplifier was designed. The origin of the noise was further discussed based on the frequency dependence of the noise levels with and without the lock-in amplifier. Our strain sensor was introduced in the Wheatstone bridge circuit.

**Figure 3.**
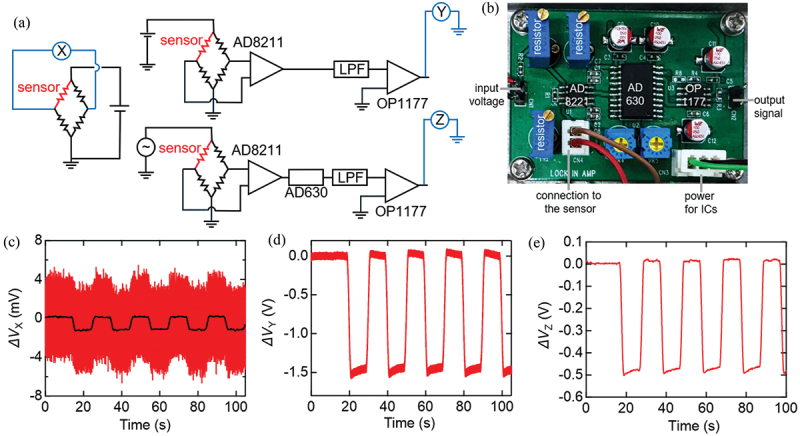
Measurements of strain signals. (a) Schematic illustrations of the circuit diagram for the Wheatstone bridge circuit, one with the amplifier (AD8221) and a low-pass filter (LPF), and the other with an additional lock-in amplifier (AD630). (b) A photo of the employed circuit board with the lock-in amplifier, whose size was 40 mm by 60 mm. (c) The measured signal at the terminal X (red). The averaged value (black) corresponding to a sample frequency of 10 Hz. DC 1 V was applied to the bridge. (d) The measured signal at the terminal Y. DC 1 V was applied to the bridge. (e) The measured signal at terminal Z. AC 1 Vpp was applied to the bridge. 230 ppm strain was applied with 10 s intervals and the sampling frequency was 1 kHz for all measurements.

The output from the bridge was directly monitored at terminal X or connected to an amplifier circuit (Figure 3(a)). Here, circuits with an amplifier and low-pass filter, or one with an additional lock-in amplifier were employed to measure the signals at terminal Y or terminal Z, respectively. The lock-in amplifier amplifies the components with a specific frequency while suppressing others, thereby reducing the influence of noise. The lock-in amplifier was constructed using the integrated circuit AD630 Analog Devices. AD630 is known for its high noise-reduction effectiveness and is used in high-precision signal processing and industrial measurement applications requiring a wide dynamic range. To achieve impedance matching, an amplifier AD8211 was inserted between the bridge and the lock-in amplifier. A low-pass filter with a cut-off frequency of 3.8 Hz and the operational amplifier OP1177 were also inserted at the output of the lock-in amplifier to stabilize the output voltage. The passive components around the IC are omitted from the description. The dimensions of the printed circuit board were as small as 60 mm × 40 mm, including three ICs and variable resistors (Figure 3(b)).

The prepared strain sensor was attached to a stainless-steel plate, using a cyanoacrylate-based adhesive CC-33A (manufactured by Kyowa Electric Co., Ltd., Japan). 230 ppm strain was applied to the plate every 10 s while measuring the output signals from the terminal X, Y, and Z by a data logger (DAQ970A, Keysight Technologies, USA). This strain was applied along the channel direction, which corresponds to the c-axis of the single crystal, as in previous studies [8,15].

The shifts in the signal voltages (*ΔV*) during repetitive application of 230 ppm strain were monitored. At the bridge terminal X (Figure 3(c)), the noise level was larger than the signals originating from the strain. Based on the time-averaged data (black line), the gauge factor was calculated to be 22, which is consistent with the reported increase in the mobility in the employed material [8]. At the bridge terminal Y (Figure 3(d)), the noise level was 11.4% as a standard deviation σ relative to the signal amplitude corresponding to 100 ppm strain. The output of the lock-in amplifier at terminal Z (Figure 3(e)) has only an output voltage with a standard deviation σ of 0.6% relative to the signal amplitude corresponding to 100 ppm strain. Using 3σ value of 1.8% as the noise in voltages, accuracy of the strain evaluation was ± 1.8 ppm.

**Figure 4.**
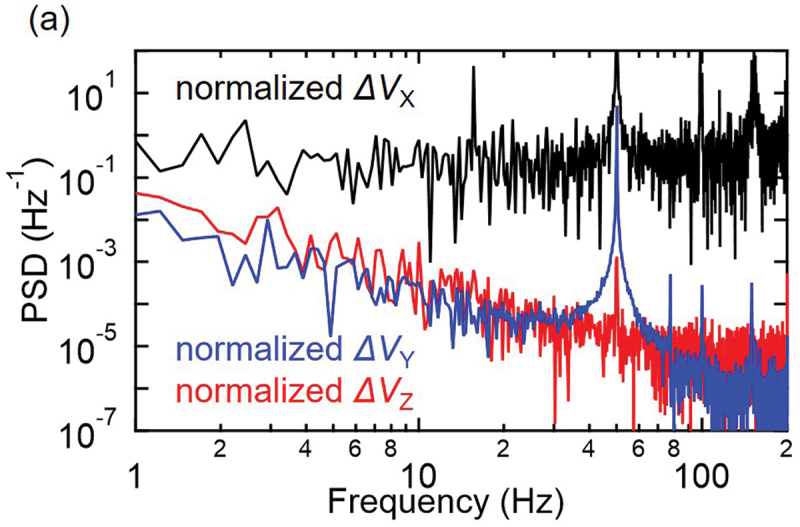
Analysis of the noise in our measurements. (a) Power spectral density evaluated for the normalized signals at terminal X (black), terminal Y (blue), and Z (red). 4096 data points were employed for this calculation.

To discuss the origin of the noise, a frequency analysis of the signals was conducted by the Fast Fourier Transformation of the measured signals. To compare the noise levels of the signals from the bridge circuit without amplification and signals from the lock-in amplifier, the signals were normalized by the amplitudes corresponding to a mechanical strain of 100 ppm. After this normalization, power spectrum density (PSD) of the signals was calculated (Figure 4(a)). Based on this result, the signals from bridge output X show a PSD independent of the frequency. Considering that the noise from the trapping events in organic semiconductors shows frequency dependence, with the noise level inversely proportional to the frequency [29], the observed noise level in our experiment may come from the measurement system and methods. When the amplifier and low-pass filter were employed at terminal Y, a significant reduction in the noise levels was confirmed over a wide frequency range. The use of the additional lock-in amplifier at terminal Z further removed noise at some specific frequencies such as 50 Hz, which may originate from the power line. Thus, in addition to the use of single crystals and chemical doping, which are advantageous for decreasing noise originating from carrier trapping in OSCs, the use of our compact circuits decreased extrinsic noise originating from the measurement system. In development of such measurement systems, it was advantageous to employ easy-to-measure, low-drift resistive sensors fabricated by stable chemical doping of OSC single crystals.

## 3. Conclusions

Strain sensors based on the C9-DNBDT-NW single crystals were fabricated using the modified ion exchange doping method. The uniform and scalable fabrication and doping process of OSC single crystals were demonstrated in a 10 cm substrate. The fabricated devices showed a conductivity of *ca*. 3 μS, suggesting that the Fermi energy is close to the valence band edge, where effective trap filling is expected. The responses of our strain sensors to mechanical strains were tested using a Wheatstone bridge circuit with a lock-in amplifier. The error in the strain measurement was estimated to be 1.8 ppm when the lock-in amplifier was employed. This demonstration of accurate strain sensing using chemically doped single crystals of OSCs and a compact lock-in amplifier highlights the importance of design in both material and measurement systems. These findings open opportunities to employ flexible strain sensors based on OSC single crystals in various sensing systems and industrial sectors.
